# Galactosylated wall teichoic acid, but not lipoteichoic acid, retains InlB on the surface of serovar 4b *Listeria monocytogenes*

**DOI:** 10.1111/mmi.14455

**Published:** 2020-03-17

**Authors:** Eric T. Sumrall, Christopher R. E. Schefer, Jeanine Rismondo, Stephan R. Schneider, Samy Boulos, Angelika Gründling, Martin J. Loessner, Yang Shen

**Affiliations:** ^1^ Institute of Food, Nutrition and Health ETH Zurich Zurich Switzerland; ^2^ Section of Microbiology and MRC Centre for Molecular Bacteriology and Infection Imperial College London London UK

**Keywords:** bacteriophages, cell wall, galactose, glycosylation, *Listeria monocytogenes*, teichoic acids

## Abstract

*Listeria monocytogenes* is a Gram‐positive, intracellular pathogen harboring the surface‐associated virulence factor InlB, which enables entry into certain host cells. Structurally diverse wall teichoic acids (WTAs), which can also be differentially glycosylated, determine the antigenic basis of the various *Listeria* serovars. WTAs have many physiological functions; they can serve as receptors for bacteriophages, and provide a substrate for binding of surface proteins such as InlB. In contrast, the membrane‐anchored lipoteichoic acids (LTAs) do not show significant variation and do not contribute to serovar determination. It was previously demonstrated that surface‐associated InlB non‐covalently adheres to both WTA and LTA, mediating its retention on the cell wall. Here, we demonstrate that in a highly virulent serovar 4b strain, two genes *gtlB* and *gttB* are responsible for galactosylation of LTA and WTA respectively. We evaluated the InlB surface retention in mutants lacking each of these two genes, and found that only galactosylated WTA is required for InlB surface presentation and function, cellular invasiveness and phage adsorption, while galactosylated LTA plays no role thereof. Our findings demonstrate that a simple pathogen‐defining serovar antigen, that mediates bacteriophage susceptibility, is necessary and sufficient to sustain the function of an important virulence factor.

## Graphical abstract

Serovar 4b strains of *L. monocytogenes* possess galactosylated WTAs and LTAs. We show that two different genes, *gtlB* and *gttB* encode two distinct glycosyltransferases required for the galactosylation of LTA and WTA respectively. Deletion of these genes reveals that the galactose decoration on WTA but not LTA is responsible for the surface retention and function of the important virulence factor InlB.

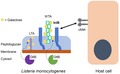

## 1 INTRODUCTION

*Listeria monocytogenes* is a Gram‐positive, facultative intracellular pathogen, causing listeriosis, a foodborne infectious disease with a significant global burden (de Noordhout et al., 2014). The ability of this bacterium to withstand high and low temperature, pH and salt concentrations (Liu, Lawrence, Ainsworth, & Austin, 2005; Radoshevich & Cossart, 2018), explains why infections are almost exclusively transmitted by contaminated food. Among the 20 designated species within the genus, the human pathogen *L. monocytogenes* can be further subdivided by serovars, a phenotypic designation relying upon the structural intricacies of the peptidoglycan‐associated wall teichoic acids which define the O‐antigens (Kamisango et al., 1982). Twelve different serovars have been described for *L. monocytogenes* (1/2a, 1/2b, 1/2c, 3a, 3b, 3c, 4a, 4b, 4c, 4d, 4e and 7) (Gasanov, Hughes, & Hansbro, 2005). More recent data indicate that the virulence‐attenuated serovar 3 and (very rare) serovar 7 strains actually originate from a serovar 1/2 background by the acquisition of small mutations in WTA biosynthesis genes, which results in loss of glycosyl modifications on the WTA backbone and serotype conversion (Eugster et al., 2015). Similarly, the virulence‐attenuated serovar 4d strains are apparently derived from a 4b background, again featuring small mutations (Sumrall et al., 2019). In this context, it is important to note that serovar 4b strains are responsible for the majority of listeriosis cases worldwide and almost all larger outbreaks (Orsi, Bakker, & Wiedmann, 2011), suggesting that strains of this type are highly virulent and possess an elevated potential to establish infection.

The Gram‐positive cell wall of *L. monocytogenes* consists of a thick layer of peptidoglycan decorated with wall teichoic acid (WTA) and lipid‐linked lipoteichoic acid (LTA). The cell wall plays a critical role in sustaining the high internal turgor pressure, as well as the rod‐like shape of the cell (Pucciarelli, Bierne, & Garcìa‐Del Portillo, 2007). The O‐antigens, which are the primary determinants of serovar, are determined by structural variations in WTA (Shen et al., 2017; Uchikawa, Sekikawa, & Azuma, 1986a). WTAs are glycopolymers covalently linked to the N‐acetylmuramic acid (MurNAc) residues of peptidoglycan, and consist of a polymeric ribitol‐phosphate chain (type I), which can also contain an integrated N‐acetyl glucosamine (GlcNAc) residue (type II). Each repeating unit in the chain is joined via phosphodiester bonds and can be glycosylated or *O*‐acetylated. It has been shown that the wide structural diversity of WTAs confers *Listeria* serovar specificity (Shen et al., 2017; Uchikawa et al., 1986a) and also determines the host range of infecting bacteriophages (Wendlinger, Loessner, & Scherer, 1996). More generally, WTAs are known to regulate cell morphology and division (Atilano et al., 2010; Bhavsar, Beveridge, & Brown, 2001; Eugster & Loessner, 2012; Soldo, Lazarevic, & Karamata, 2002), autolytic activity (Schlag et al., 2010; Wecke, Madela, & Fischer, 1997) and cation homeostasis (Biswas et al., 2012; Swoboda, Campbell, Meredith, & Walker, 2010). They are also involved in the presentation of envelope proteins (Carvalho, Sousa, & Cabanes, 2018), protection from host defenses and antibiotics (Collins et al., 2002; Farha et al., 2013; Kristian et al., 2005; Weidenmaier et al., 2004; Xia, Kohler, & Peschel, 2010), and mediation of host invasion and colonization (Walter et al., 2007; Weidenmaier et al., 2004). Moreover, the LTA molecules are associated with the bacterial membrane via a glycolipid anchor and consist of glycerol phosphate repeating units substituted with galactose (Gal) and d‐alanine residues (Brown, Santa Maria, & Walker, 2013; Campeotto et al., 2014; Neuhaus & Baddiley, 2003). Together with the peptidoglycan and WTAs, the LTAs contribute to a polyanionic network that plays a key role in cellular fitness and virulence (Percy & Gründling, 2014). Although both WTAs and LTAs are zwitterionic polymers, their biosynthesis proceeds through separate pathways, which have been well‐characterized and investigated as potential antibacterial targets (Sewell & Brown, 2014). Unlike WTAs, the LTAs do not show much structural diversity amongst the *L. monocytogenes* serovars (Hether & Jackson, 1983; Uchikawa, Sekikawa, & Azuma, 1986b), and there is no evidence that they are involved in determining serovar identity. The WTA of serovar 4b strains features a type II structure, with a GlcNAc moiety integrated into the chain, which is further decorated by galactose, glucose and *O*‐acetyl groups (Shen et al., 2017; Uchikawa et al., 1986a). The high level of virulence of serovar 4b isolates (compared to genetically similar strains) was shown to be directly dependent on its specific galactosylated structure (Sumrall et al., 2019).

Since the cell wall represents the outermost layer of the Gram‐positive cell, it is responsible for interacting with the environment, which in the case of *L. monocytogenes*, can be the mammalian host. Many proteins known to be involved in the crossing of host barriers are associated either covalently or non‐covalently with the cell wall (Cabanes, Dehoux, Dussurget, Frangeul, & Cossart, 2002). Internalin B (InlB) is an invasin required for entry into certain mammalian cells and functions by hijacking the receptor‐mediated endocytosis machinery via the host receptors cMet and gC1QR (Bleymüller et al., 2016; Braun, Ghebrehiwet, & Cossart, 2000; Braun, Ohayon, & Cossart, 1998; Khelef, Lecuit, Bierne, & Cossart, 2006; Shen, Naujokas, Park, & Ireton, 2000). Initially, it was thought that InlB is non‐covalently associated with the bacterial cell wall by interacting with the LTA molecules via its C‐terminal Csa domain, which features a canonical GW motif (Jonquieres et al., 1999). More recently, this assumption was questioned by the observation that other Csa‐domain‐containing proteins are retained in the cell wall in the absence of LTA (Percy, Karinou, Webb, & Gründling, 2016), which also suggested that another ligand may be involved in the surface binding and presentation of InlB. Recent data from our lab revealed that loss of the galactose decoration on both LTA and WTA in serovar 4b strains led to a complete loss and secretion of InlB, and an inability of this galactose‐deficient mutant to perform InlB‐dependent cellular invasion (Sumrall et al., 2019). While it was shown that purified InlB can bind to both WTA and LTA polymers in a galactose‐dependent manner, it was not clear whether LTA or WTA galactosylation (or both) is responsible for retention and display of InlB on the cell wall.

In this study, we have identified two different glycosyltransferases, one involved in the galactosylation of LTA, and the other in the galactosylation of WTA. This allowed us to evaluate the role of Gal‐LTA and Gal‐WTA separately. Our data clearly demonstrate that galactosylated WTA is the sole mediator of InlB surface retention, whereas LTA plays no role thereof.

## 2 RESULTS

### 2.1 Galactosylation of LTA and WTA is mediated through separate pathways

**Figure 1:**
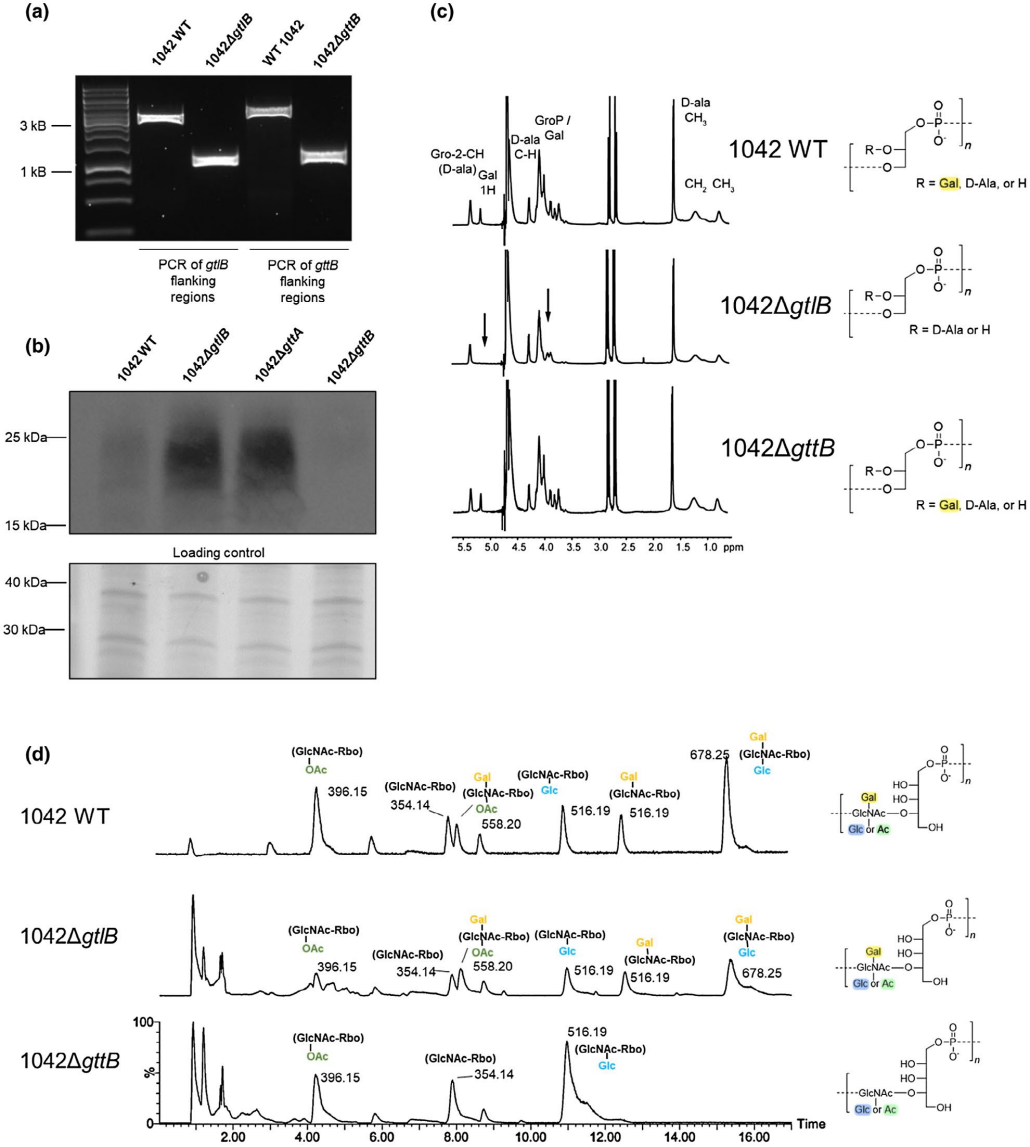
Galactosylation of LTA and WTA in a serovar 4b strain is controlled by two separate genes. (a) Agarose gel electrophoresis of PCR‐amplified regions flanking genes *gtlB* and *gttB* from WT 1042 and the 1042Δ*gtlB* and 1042Δ*gttB* mutant strains. Primers used for the PCR amplification recognize sequences flanking the genes of interest by ~ 600 bp. (b) LTA analysis by western blot. Whole cell extracts were separated on SDS‐PAGE gels and LTA detected using an LTA‐specific antibody, which recognizes the glycerol phosphate backbone of LTA. Positive signal represents undecorated LTA. Lower panel: SDS‐PAGE gel visualization of total extracts to demonstrate equal loading. (c) NMR spectra of the LTA isolated from the indicated strains. Labeled peaks represent the major protons in the sample, while missing galactosylated protons are indicated with arrows. The LTA structure for each strain is indicated to the right. The unlabeled major peaks are derived from the residual citrate buffer used during the extraction process. (d) Liquid chromatographic separation and MS‐based identification of WTA monomer residues derived from WT 1042 and the indicated mutants. Major peaks are labeled with their assigned structures based on the *m/z*. The chromatograms are aligned on the same time axis to allow for comparison. Peaks appearing at 1–2 min represent ionized or undigested species that elute without separation

We have previously shown that loss of galactose on both WTA and LTA leads to the release of InlB from *Listeria* cell surfaces, indicating a functional relationship between TA and InlB (Sumrall et al., 2019). In order to evaluate the specific effects of Gal‐LTA and Gal‐WTA on the retention of InlB, we created a knockout in *gtlB*, which had previously been identified as mediating galactose decoration of the LTA chain in a serovar 1/2 background (Rismondo, Percy, & Gründling, 2018). Because the type I WTA in serovar 1/2 is substituted with GlcNAc and rhamnose, and not Gal, we hypothesized that in serovar 1/2 the *gtlB* product may actually act on the LTA chain (i.e. function as a LTA‐specific galactosyltransferase), otherwise the WTA would likely exhibit some galactosylation. If GtlB in a serovar 4b strain carries the same specificity, a *gtlB* deletion would leave the Gal moiety present on this mutant’s WTA intact, removing only the LTA’s Gal moiety. The gene *gttB*, immediately downstream of the previously identified *gttA* (Sumrall et al., 2019), encodes a putative membrane‐associated protein homologous to known PMT‐family glycosyltransferases. No homologue is present in the serovar 1/2 strains 10403S or EGD‐e, indicating that it represents a serovar 4b WTA‐specific glycosyltransferase. Therefore, *gtlB* and *gttB* were deleted in the serovar 4b strain 1042, resulting in strains 1042Δ*gttB* and 1042Δ*gtlB*. All mutations were confirmed by PCR and sequencing (Figure 1a).

To determine whether these knockouts impact the glycosylation pattern of LTA in the serovar 4b background, a western blot was performed using an anti‐LTA antibody, which recognizes the glycerol phosphate backbone. It was previously demonstrated that the anti‐LTA signal increases when LTA lacks a glycosidic substitution, presumably because the glycerol‐phosphate backbone of LTA becomes more accessible (Percy et al., 2016; Rismondo et al., 2018). Indeed, the anti‐LTA signal increased in a western blot using cell extracts derived from strain 1042Δ*gtlB* (Figure 1b). This is similar to the increase in signal as observed for strain 1042Δ*gttA,* which we have previously shown lacks Gal residues on its LTA polymers (Sumrall et al., 2019). Moreover, no change in signal compared to wild type was seen for strain 1042Δ*gttB*, suggesting that the LTA glycosylation status is unchanged in this mutant (Figure 1b). To confirm this result, the LTA polymer was purified from cultures and analyzed by 1D ^1^H NMR. Obtained proton spectra from 1042 WT as well as 1042Δ*gttB* indicated the expected glycerol‐phosphate structure, substituted with both d‐alanine and α‐galactose. However for 1042Δ*gtlB*, the peaks representing the galactosylated species were missing (Figure 1c), indicating that only GtlB and not GttB is required for the galactosylation of LTA. Although biochemical evidence is lacking, one can assume that GtlB functions in serovar 4b as previously established for serovar 1/2.

To evaluate the WTA status in these two mutants, we purified and HF‐depolymerized the type II WTAs using a HPLC‐MS/MS‐based methodology (Shen et al., 2017). We identified the repeating unit structure based on the retention time and mass‐charge ratio (*m/z*). Fragmentation patterns for the fully glycosylated 1042 serovar 4b WTA demonstrated the ribitol‐phosphate‐GlcNAc backbone structure with galactose (Gal), glucose (Glc) and *O*‐acetyl (*O*Ac) decorations on the integrated GlcNAc residues (Shen et al., 2017). As hypothesized, the 1042Δ*gtlB* mutant reveled no WTA structural change, while 1042Δ*gttB* lacked the *m/z* 558.20 (~8.1 min) signal representing the *O*‐acetylated Gal‐GlcNAc‐Rbo species, the later‐eluting *m/z* 516.20 (~12.7 min) signal denoting the Gal‐GlcNAc‐Rbo fragment, and the signal at 678.25 *m/z* eluting around 15 min corresponded to the GlcNAc‐Rbo repeating unit substituted by both Gal and Glc. These data confirm that the WTA derived from strain 1042Δ*gttB* lacks the Gal decoration, but still possesses the Glc and *O*Ac decorations, thus sanctioning a serovar 4d designation (Figure 1d). Together, these data confirm our hypothesis that serovar 4b strains, which normally possess a Gal decoration on both LTA and WTA molecules, possess two different surface‐acting galactosyltransferases with distinct substrate specificity.

### 2.2 Bacteriophages with a 4b‐specific host range require only Gal‐WTA for adsorption

**Figure 2:**
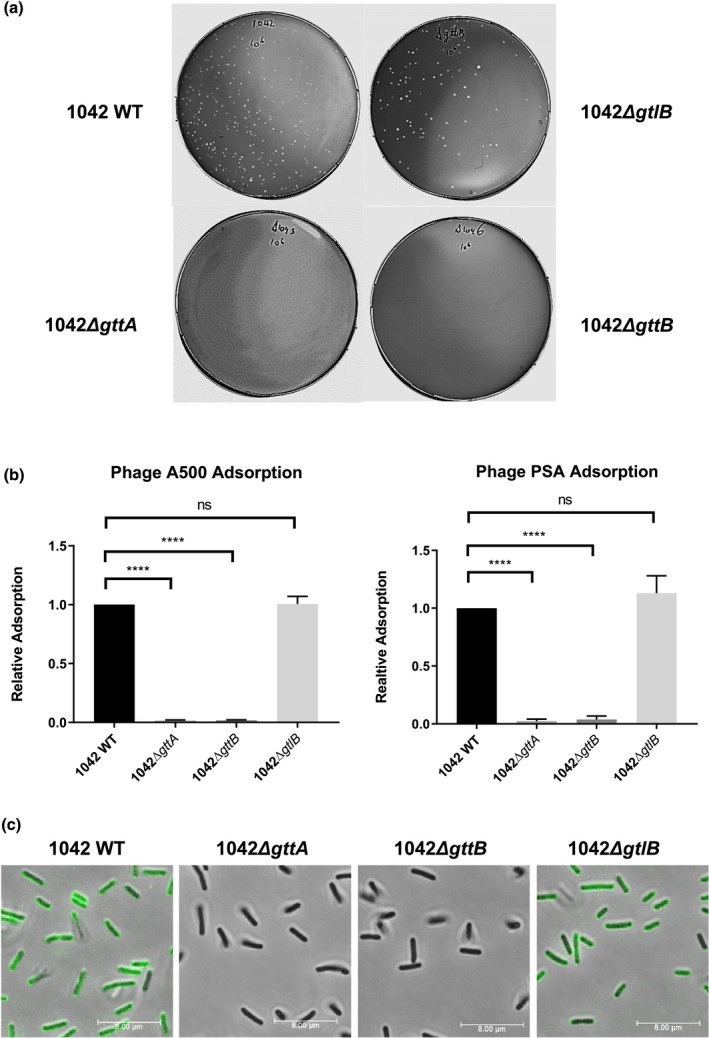
WTA galactosylation, but not LTA galactosylation, mediates the phage adsorption in *Listeria monocytogenes* serovar 4b. (a) Phage overlay experiments using the serovar 4b‐specific bacteriophage A500 and infecting the indicated host strains. (b) Relative adsorption of the serovar 4b‐specific phages A500 and PSA to the indicated strains, compared to WT 1042, as determined by a phage pulldown assay (bars represent mean ± *SD*; ****p < .0001, ns = not significant; *n* = 3). (c) Staining of the indicated strain with the recombinant phage A500 Gp19 receptor binding protein fused to GFP, and visualized by fluorescence confocal microscopy

Bacteriophages A500 and PSA possess the unique ability to adsorb to and infect serovar 4b strains (Dorscht et al., 2009; Loessner & Busse, 1990; Zink & Loessner, 1992). Deletion of *gttA* resulted in resistance to A500 and PSA infection, due to loss of adsorption (Dunne et al., 2019; Sumrall et al., 2019). Nevertheless, it could not be definitively concluded whether these phages utilize Gal‐WTA alone as a receptor, since GttA mediates the addition of Gal to both LTA and WTA chains. Plaque assays with phage A500 using the two newly constructed mutant strains 1042Δ*gttB* and 1042Δ*gtlB*, as well as 1042Δ*gttA* and 1042 WT as negative and positive controls respectively, showed that loss of the Gal moiety from WTA is sufficient to confer phage resistance (Figure 2a). We conclude that this is due to loss of the phage‐binding receptor, as adsorption of both A500 and PSA phages to the cell surface of strain 1042Δ*gttB* was abolished, while adsorption to the cell surface of strain 1042Δ*gtlB* remained unchanged (Figure 2b). Consistent with this, the *gp19*‐encoded receptor‐binding protein of phage A500 (unpublished data) fused to GFP was unable to bind to the cell surface of strains deficient in Gal‐WTA, while affinity for the 1042Δ*gtlB* strain deficient in Gal‐LTA showed no change relative to WT, as assessed by fluorescence microscopy (Figure 2c). The specific affinity of 4b‐specific phages and their receptor binding protein for Gal‐WTA, and not Gal‐LTA, indicates that Gal‐WTA alone mediates the 4b specificity of these phages.

### 2.3 Galactosylated WTA, not LTA, retains InlB to the surface of serovar 4b *L. monocytogenes*

**Figure 3:**
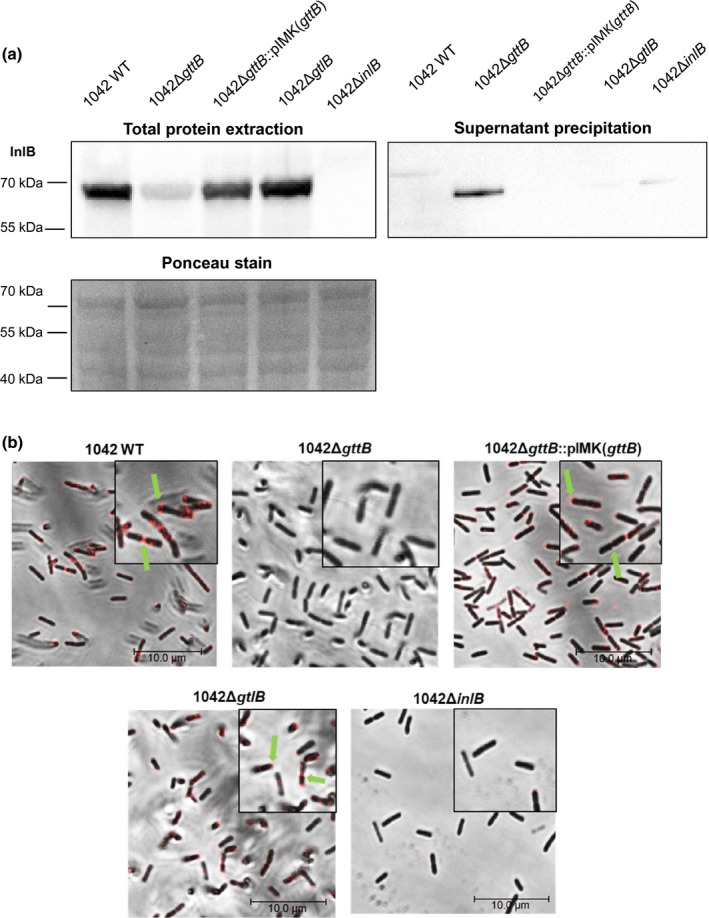
InlB surface retention is dependent upon WTA galactosylation but not LTA galactosylation. (a) Western blot analysis using total protein (left) and precipitated supernatant (right) fractions derived from equal cell numbers of the indicated strains. InlB was detected using an anti‐InlB antibody. Ponceau staining of the PVDF membrane is provided to indicate equal loading. Samples were normalized for OD and protein concentrations were further normalized before loaded onto an SDS‐PAGE gel. (b) Immunofluorescence using the indicated strains and an anti‐InlB polyclonal antibody (Red). Insets contain a zoomed portion of the image. Green arrows indicate the polar localization of InlB localized on the bacterial surface

Recent findings highlighted that InlB is retained to the cell surface when teichoic acids are galactosylated (Sumrall et al., 2019). However, it remained unclear whether LTA and WTA function synergistically, or if one molecule plays a more significant role. Western blot of total cellular proteins demonstrates that the 1042Δ*gtlB* cell wall retains an equal amount of InlB compared to the wild type (Figure 3a). Moreover, a significant decrease in cell‐associated InlB was observed in the 1042Δ*gttB* mutant*,* with a concomitant increase of InlB secreted into the supernatant (Figure 3a). This loss of InlB surface localization was equivalent to that in the *gttA* mutant (Supplementary Figure S1), which mediates galactosylation of both WTA and LTA (Sumrall et al., 2019).This phenotype was complemented by expressing *gttB* from an integrative plasmid (Monk, Gahan, & Hill, 2008), although InlB did not return entirely to WT levels, likely due to incomplete complementation (Figure 3a). Immunofluorescence of whole cells further demonstrates the dependence of InlB surface presentation on Gal‐WTA, but not Gal‐LTA (Figure 3b). The polar localization of InlB remained unaffected in the 1042Δ*gtlB* strain but was absent in the 1042Δ*gttB* mutant, which could again be reconstituted by trans‐complementation.

**Figure 4:**
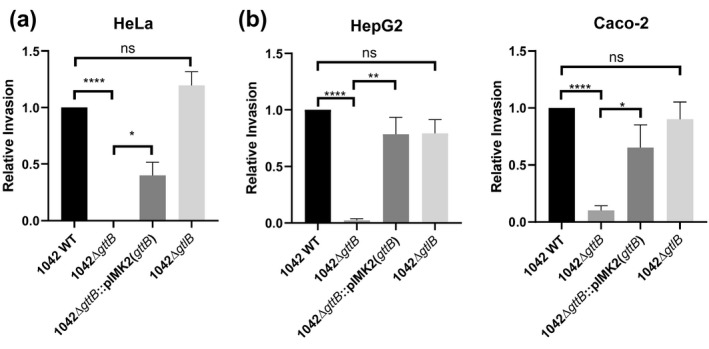
Loss of WTA galactosylation leads to attenuation of cellular invasiveness. (a) Relative invasiveness of the indicated strains compared to 1042 WT, as determined via a 3 hour infection of HeLa cells, for which invasion is primarily mediated by InlB (mean normalized to WT ± *SEM*, ****p < .0001, *p < .05; *n* = 3). (b) Relative invasiveness of the same strains as in (a) compared to 1042 WT, as determined via a 3 hour infection of Caco‐2 (*n* = 4) and HepG2 cells (*n* = 3) (mean normalized to WT ± *SEM*, ****p < .0001, **p < .01, *p < .05, ns = not significant)

In order to investigate the extent to which loss of InlB surface presentation affects the cellular invasion, we performed the invasion assays using HeLa cells, which do not express E‐cadherin (Vessey et al., 1995), meaning that invasion is exclusively mediated by InlB. The invasion rate of the 1042Δ*gttB* knockout dropped to near‐zero (Figure 4a), indicating that cells lacking the Gal substitution on their WTA do not possess the ability to retain InlB and activate the cMet‐mediated invasion pathway. Again, this phenotype could be complemented upon expression of *gttB* from an integrative plasmid. No difference in the invasion rate was seen for strain 1042Δ*gtlB* compared to the WT strain (Figure 4a), although it did not reach WT levels, likely again due to incomplete complementation of *gttB*. Similar results were observed when using HepG2 and Caco‐2 cell lines, where invasion is also predominately dependent upon InlB; a significantly attenuated invasion rate was observed for strain 1042Δ*gttB*, but not with 1042Δ*gtlB* (Figure 4b). We previously showed that the InlA surface expression (Sumrall et al., 2019) is unaffected by the galactosylation of both LTA and WTA in this strain, meaning the invasion reduction observed here is mainly mediated by loss of InlB retention. Together, these data highlight that Gal‐WTA alone is necessary and sufficient to retain and display InlB at the cell surface, and that Gal‐LTA does not play a measurable role in presenting of InlB.

## 3 DISCUSSION

The data presented here demonstrate that WTA, but not LTA, is the primary mediator of InlB surface retention and function. The finding that WTA and LTA are galactosylated by two different enzymes allowed us to investigate the biological function of the galactose modification on WTA and LTA separately. While we did not observe any recognizable difference in growth rate between the wild‐type strain and the *gtlB* or *gttB* mutants (Supplementary Figure S2), we found that cellular invasiveness is impaired only in the galactose‐deficient WTA mutant, due to loss of InlB.

**Figure 5:**
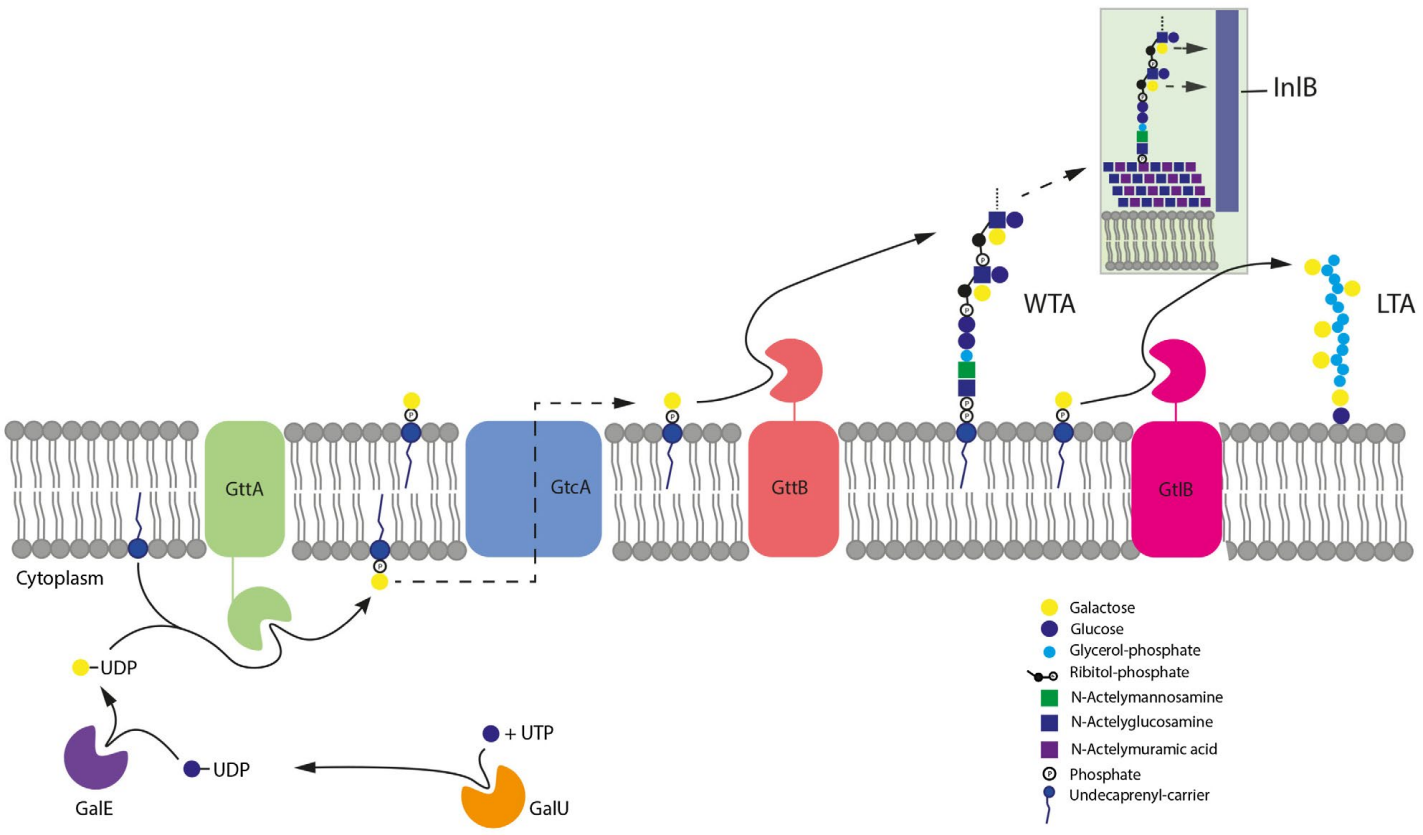
Updated model for teichoic acid galactosylation pathways in *L. monocytogenes* serovar 4b. Starting from the beginning of the pathway, GalU (a glucose‐1‐phosphate uridyltransferase) produces UDP‐Glc from UTP and glucose‐1‐phosphate. Next, GalE (a UDP‐glucose 4‐epimerase) catalyzes the conversion of UDP‐Glc to UDP‐Gal. GttA next catalyzes the addition of Gal onto the UndP lipid carrier molecule on the inner leaflet of the cell membrane. The UndP‐Gal is flipped by the putative flippase GtcA to the outer leaflet of the membrane, creating a pool of lipid‐linked Gal to be added onto either WTA or LTA chains. GttB catalyzes the addition of Gal from UndP‐Gal onto the growing WTA chain, while GtlB performs the analogous reaction and transfers Gal onto the growing LTA chain. The LTA chain remains embedded in the membrane, while the WTA chain is conjugated by TarTUV homologue to the MurNAc of peptidoglycan following export by an ABC transporter. UDP‐Glc is likely also used by GltB (not to be confused with GtlB) to decorate WTA with Glc, as it has been shown that deletion of *galU* or *gtlB* leads to a loss of WTA Glc decoration (Sumrall et al., 2019)

These findings enabled us to update the current model for teichoic acid galactosylation in *L. monocytogenes* (Figure 5). A WTA galactosylation pathway has been proposed based on findings using a serovar 4c strain (Spears et al., 2016), and our data now provide experimental evidence for the role of the individual gene products involved in WTA galactosylation (including *galU*, *galE*, *gttA* and *gttB*) in serovar 4b strains. *gttB* is located in a two‐gene operon together with *gttA,* the latter of which is proposed to be responsible for transferring UDP‐galactose to the lipid carrier, undecaprenol‐phosphate (P‐C_55_). The product of *gttB* is predicted to be a membrane protein harboring a putative glycosyltransferase domain from the PMT family (PF02366). Deletion of *gttB* resulted in loss of the galactose modification on WTA (Figure 1), allowing us to propose that GttB in fact catalyzes the addition of Gal onto the integrated GlcNAc residue of only the WTA repeating units (Figure 5).

The suggestion that LTA may be involved in retaining InlB to the surface was based on the finding that the protein was shown to bind to purified LTA in in vitro assays (Jonquieres, et al., 1999). While we have been able to confirm this observation, we showed that such binding is dependent on the presence of galactose, which when lost, drastically reduces the affinity of the Csa domain of InlB for LTA (Sumrall et al., 2019). However, we also found that InlB adheres to WTA polymers, also in a galactose‐dependent manner. In fact, this specific dependency seems more pronounced, since Gal‐depleted WTA showed no detectable affinity for InlB (Sumrall et al., 2019). This suggested that LTA may not be the actual mediator of InlB surface retention. In fact, cells lacking the LTA galactose or d‐Ala decorations, or the entire LTA molecule, were still able to retain Csa‐domain (GW‐motif)‐containing proteins (Percy et al., 2016). Similarly, it was previously reported that in serovar 1/2 cells, Csa‐containing proteins also bind to WTA polymers, in this case dependent on presence of rhamnose (Carvalho et al., 2018). Here, we show that the absence of the galactose decoration on WTA, but not on LTA, leads to release of InlB from the bacterial cell surface (Figure 3). Since the Gal decoration on LTA was shown to be heavily responsible for the direct interaction with InlB when evaluated using the purified molecules (Sumrall et al., 2019), we can conclude that LTA plays no role in the retention of InlB, or any Csa domain‐containing protein for that matter.

Given the previous indication that InlB can bind to LTA (Jonquieres et al., 1999), the question arises why this seemingly strong interaction does not function to retain InlB to the cell surface in a physiological setting. A likely explanation could be the poor accessibility of LTA to the Csa domain of InlB. This is supported by the indication that LTA might not be exposed on the cell surface (Reichmann & Gründling, 2011). In this context, it was recently shown that the full length LTA polymer contains only around 13 glycerol‐phosphate units (which interestingly elongated to around 20 units upon deletion of *gtlB*) (Percy et al., 2016; Rismondo et al., 2018). Because the Csa domain of InlB lies at the C‐terminus, and the membrane‐spanning portion of the protein is at the N‐terminus, it is possible that the LTA chain is not long enough to extend toward and interact with the Csa domain of InlB. WTA polymers moreover, are estimated to possess a longer chain length ranging from 20 to 40 units (Uchikawa et al., 1986a), and unlike LTAs, do not begin at the cytoplasmic membrane, but instead are covalently conjugated to the peptidoglycan of cell walls. Therefore, the WTAs are able to extend above the cell surface and should be accessible from the outside. They can be recognized by, and interact with, antibodies and bacteriophages. Thus, it seems obvious that WTAs are also much better suited to interact with Csa‐domain‐containing proteins than the ‘buried’ LTAs. This situation is in agreement with data showing that purified LTA can detach InlB from the cell surface, and that purified InlB is still associated with *Listeria* l‐form cells (which do not possess a cell wall, but still present LTA) (Dell'Era et al., 2009; Jonquieres et al., 1999).

How exactly glycosylated WTA affects the function of other proteins remains to be determined. Many other proteins, including the virulence‐modulating factors Ami and Auto, also feature Csa domains, and thus depend on WTA for their surface presentation (Carvalho et al., 2018). Similarly, a serovar 4c strain that lacks Gal from WTA was shown to have a defect in actin tail formation (Spears et al., 2016), although the LTA structure was not determined in these mutants.

The fact that glycosylation of WTA has seemingly broad implications for overall fitness and virulence in *L. monocytogenes*, turns our interest toward bacteriophages, whose interaction with host cells is specifically dependent on recognition of WTA structure. Given that loss of WTA glycosylation renders the bacterium insensitive to phage attack (Eugster et al., 2015; Sumrall et al., 2019; Wendlinger et al., 1996), one can presume that some selective pressure must exist that causes the bacterium to maintain this glycosylation, despite the clear disadvantage of being a target for phage. The maintenance of virulence factor function could present such selective pressure, as the ability to survive in a host could, in certain circumstances, trump the advantageousness of phage resistance. An analogous situation has also been observed in other bacteria. For example in *Salmonella*, where phase variation of O‐antigen chain length potentiates the bacterium to be either phage resistant or virulent (Cota et al., 2015). This study showed that the effect was epigenetically regulated, and therefore only transient. In our case, the acquisition of phage resistance is likely permanent. WTA glycosylation has already been shown to confer certain other advantages to the cell, such as resistance to antimicrobial peptides (Carvalho et al., 2015), cold tolerance (Chassaing & Auvray, 2007) and general virulence (Autret, Dubail, Trieu‐cuot, Berche, & Charbit, 2001; Spears et al., 2016, 2008; Sumrall et al., 2019). Now, a clearer picture emerges, showing that *L. monocytogenes*, and likely other bacteria, must balance maintaining certain types of fitness against susceptibility to bacteriophage predation. Other examples exist where loss of a phage binding receptor leads to attenuated virulence in the bacterium (Capparelli et al., 2010; Heierson, Siden, Kivaisi, & Boman, 1986; Laakso et al., 2011; Laanto, Bamford, Laakso, & Sundberg, 2012; Mori, Fukunaga, Shimamura, Nakai, & Park, 2002; Santander & Robeson, 2007). Altogether, it seems relevant to consider that bacteriophages, which are increasingly being used as biocontrol agents, may function not only as antimicrobials, but also as antivirulence agents. Future studies will focus on unraveling the precise roles of WTA galactosylation in other contexts, namely bacterial physiology, antibiotic action, host‐mediated response, adhesion, biofilm formation and virulence.

## 4 EXPERIMENTAL PROCEDURES

### 4.1 Bacterial strains, plasmids, phages and growth conditions

All bacterial strains, plasmids and phages used in this study are listed in Tables S1–S3. *E. coli* XL1‐Blue (Stratagene) used for cloning and plasmid construction was routinely cultured in Luria–Bertani (LB) broth at 37°C. *L. monocytogenes* strains were grown in 1/2 brain heart infusion (BHI), at 30°C with shaking when working with phages, or at 37°C when the cells were used for infection studies. *L. monocytogenes* mutants were constructed in a WSLC1042 background (Klumpp et al., 2014). For the selection of plasmids, growth medium was supplemented with antibiotics as indicated: ampicillin (100 μg/ml for *E. coli*), erythromycin (300 μg/ml for *E. coli*, 10 μg/ml for *L. monocytogenes*), kanamycin (50 μg/ml). Propagation of bacteriophages A500 and PSA was performed using *L. monocytogenes* strain WSLC1042, and purification was performed as previously described (Klumpp et al., 2008; Lauer, Chow, Loessner, Portnoy, & Calendar, 2002). Phage stocks were stored at 4°C in CsCl and diluted into SM Buffer (100 mM NaCl, 8 mM MgSO_4_, 50 mM Tris‐HCl, pH 7.5) before use. Host strains were grown overnight in sterile‐filtered 1/2 BHI broth for adsorption and infection assays. For growth curve determination, overnight cultures were diluted in full BHI to an OD of 0.01 in triplicate in a 96‐well plate, and the OD_600_ was measured for 10 hr on a plate reader set to 37°C.

### 4.2 *L. monocytogenes* knockout construction and complementation strains

Deletion strains were constructed as previously described (Sumrall et al., 2019), using the allelic exchange vector pHoss1 (Abdelhamed, Lawrence, & Karsi, 2015). Primers used for production of the gene deletion strains and for confirmation of the knockout strains are listed in Supplementary Table S1. Sanger sequencing confirmed complete deletion of *gtlB* and *gttB* from the genome. For construction of the complementation strain 1042Δ*gttB*::pIMK2(*gttB*), the *gttB* gene was inserted by Gibson assembly into plasmid pIMK2 under control of the P_help_ promoter (Monk et al., 2008). Re‐introduction of the *gttB* gene was confirmed by PCR (see Table S1 for primer list), and re‐expression of the WTA‐Gal moiety was confirmed by Gp19‐GFP binding.

### 4.3 Production of A500 Gp19‐GFP and fluorescence microscopy

The Gp19 protein from phage A500 was initially identified based on sequence homology to receptor‐binding proteins from other known phages, and expressed from the IPTG‐inducible pQE‐30 vector (Thermo‐Fisher) containing hGFP cloned into the BamHI and SacI sites immediately downstream of the His‐tag (pHGFP), as performed previously with bacteriophage CBDs using purified phage DNA as PCR template (Schmelcher et al., 2010). The *gp19* from A500 (Dorscht et al., 2009) sequence without the start codon was integrated into the SacI and SalI restriction sites immediately downstream of the *hgfp* sequence resulting in the construction of plasmid pGp19‐GFP allowing the expression of a Gp19‐GFP fusion protein. Protein purification was performed and evaluated as previously described for FP‐CBDs using a nickel‐NTA resin column (Schmelcher et al., 2010). Galactose dependency was evaluated by comparing protein binding between 1042 WT and 1042Δ*gttA*. Fluorescence microscopy to evaluate cell‐wall binding was performed as previously described for bacteriophage CBDs (Lauer et al., 2002). For InlB surface immunostaining of *L. monocytogenes*, cells were prepared and stained as previously described (Sumrall et al., 2019).

### 4.4 Phage overlays and adsorption analysis

Plaque assays using bacteriophage A500 and adsorption analysis using A500 and PSA was performed as previously described, using WSLC 1042 as prey (Wendlinger et al., 1996). Following serial dilutions of the phage, the number of phages adsorbed to the bacterial surface was evaluated by the plaque assay and expressed as the relative PFU of phage adsorbed to the mutant, compared to that of the WT.

### 4.5 WTA and LTA extraction and structural analysis

Cell walls from *L. monocytogenes* strains were prepared for extraction and purification of WTAs as previously described (64). Digested WTA monomers were subjected to UPLC‐MS/MS for compositional and structural analysis, as previously described (Shen et al., 2017). LTA was extracted and analyzed as previously described (Gründling & Schneewind, 2007; Percy et al., 2016). Determination of LTA galactosylation by western blot was performed also as previously described (Percy et al., 2016), using a polyglycerolphosphate‐specific antibody and whole‐cell extracts (Clone 55 from Hycult biotechnology). Determination of equal loading was done by imaging the extracts on a Stain‐Free Criterion TGX gel before membrane transfer.

### 4.6 InlB detection by Western blot

To detect total InlB, total cell extracts were used instead of surface protein extracts in order to obtain a better visualization for the loading control. One mL of overnight culture grown in 1/2 BHI medium was mixed with 0.5 ml of 0.5 mm glass beads in a 2 ml tube and shaken on a vortex at maximum speed for 30 min. The tubes were spun down for 1 min at 200 × g and 500 μl of supernatant was transferred to a 1.5 ml Eppendorf, and spun again for 15 min at 16,100 g speed. The supernatant was discarded and the pellet resuspended in 50 μl SDS sample buffer for an initial OD of 2 (volume was adjusted accordingly if initial OD varied) containing 5% β‐mercaptoethanol, and boiled for 5 min. Protein extract concentrations were measured using the BCA Protein assay kit (Pierce Biotechnology), and 10 μg of sample was loaded onto an SDS‐PAGE gel and western blot was performed as previously described (Sumrall et al., 2019), using a custom anti‐InlB rabbit polyclonal antibody (1:5,000). PVDF membranes were stained with Ponceau and imaged to further demonstrate equal loading. Supernatant extracts were produced via a TCA precipitation method using the supernatant from a 5 ml culture, as previously described (Kocks et al., 1992) and blotted using the same anti‐InlB polyclonal antibody.

### 4.7 Cell culture techniques

The Caco‐2 (ATCC HTB‐37), HepG2 (ATCC HB‐8065) and HeLa cell lines (Sigma‐Aldrich, 93021013) used for in vitro assays were cultured at 37°C with 5% CO_2_ in DMEM GlutaMAX (Gibco), supplemented with sodium pyruvate, 1% non‐essential amino acids and 10% FBS. For Caco‐2 and HeLa cell lines, 4.5 g/L of glucose was used; for HepG‐2 cells, 1 g/L was used. Invasion assays (gentamicin protection assays) were performed as previously described (Sumrall et al., 2019). The number of bacteria invaded was expressed as a fraction relative to that of WT strain 1042. For all in vitro experiments, at least three biological replicates were performed, each time with three technical replicates.

## AUTHOR CONTRIBUTIONS

Conception and design: ETS, YS. Acquisition, analysis and interpretation of the data: ETS, CS, JR, SB, SRS, YS, AG, MJL. Writing of the manuscript: ES. Reviewing and editing of the manuscript: JR, AG, YS, MJL.
