# Supplementary material for: Galactosylated wall teichoic acid, but not lipoteichoic acid, retains InlB on the surface of serovar 4b Listeria monocytogenes
Source: Mol Microbiol. 2020 Mar 17;113(3):638–49. doi: 10.1111/mmi.14455 (PMC7155027; doi:10.1111/mmi.14455)
Supplement: Supplementary file 1 [file MMI-113-638-s001.docx]

**Supplementary Information**

**Galactosylated wall-teichoic acid, but not lipoteichoic acid, retains InlB on the surface of serovar 4b *Listeria monocytogenes***

Eric T. Sumrall^1^, Christopher R.E. Schefer^1^, Jeanine Rismondo^2^, Stephan R. Schneider^1^, Samy Boulos^1^, Angelika Gründling^2^, Martin J. Loessner^1*^, and Yang Shen^1*^

*^1^ Institute of Food, Nutrition and Health, ETH Zurich, Schmelzbergstrasse 7,
CH-8092 Zurich, Switzerland*

*^2^ Section of Microbiology and MRC Centre for Molecular Bacteriology and Infection,
Imperial College* *London, London, UK*

* For correspondence. E-mail [martin.loessner@ethz.ch](mailto:martin.loessner@ethz.ch) or yang.shen@hest.ethz.ch; Tel +41 44 632 3335.

**Running title:** *Listeria* wall teichoic acid retains InlB

**Keywords:** *Listeria monocytogenes*, Cell Wall, Teichoic Acids, Glycosylation, Galactose, Bacteriophages

**
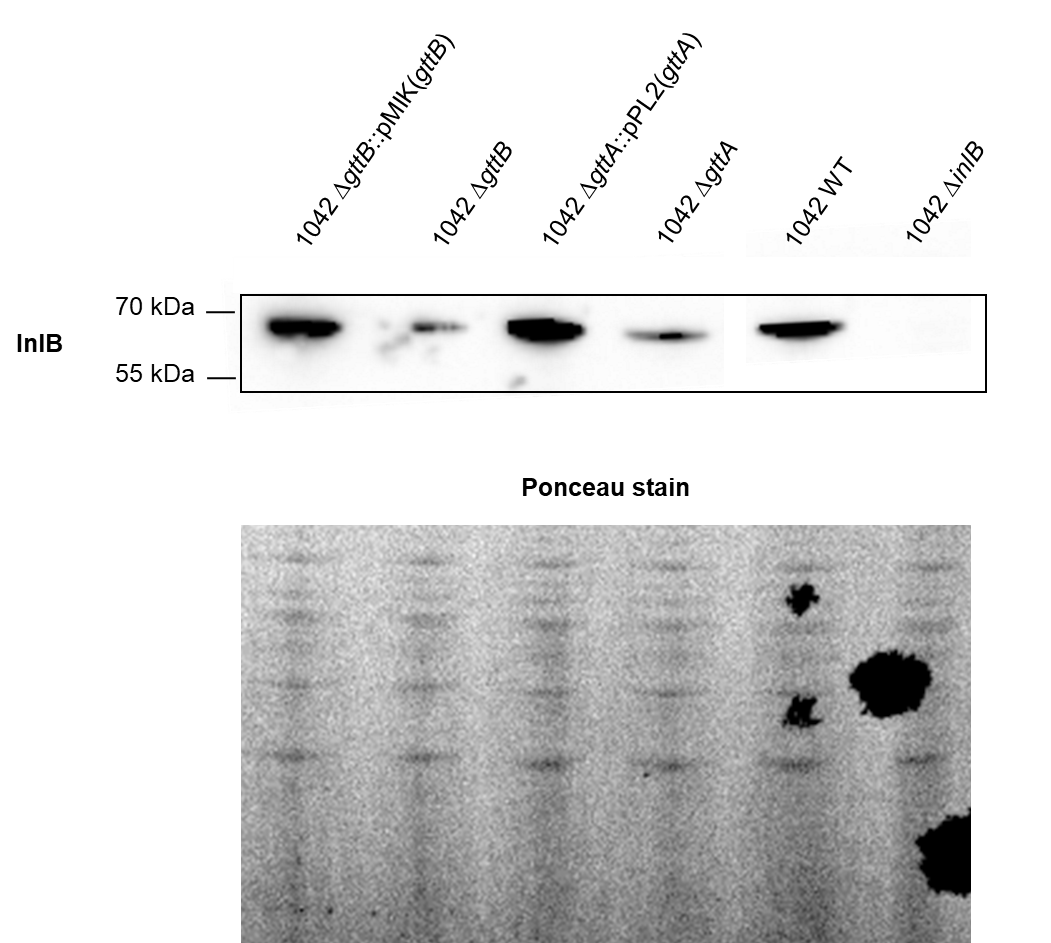
**

**Supplementary Figure S1. Western blot analysis using total protein fractions derived from equal cell numbers of the indicated strains.** InlB was detected using an anti-InlB antibody. Ponceau staining of the PVDF membrane is provided to indicate equal loading. Samples were normalized for OD and protein concentrations were further normalized before loaded onto an SDS-PAGE gel.

**Supplementary
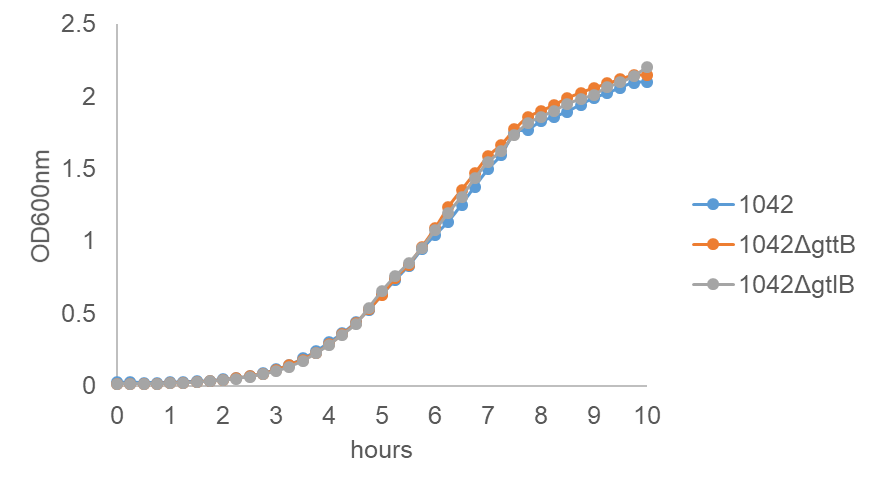
Figure S2. Growth curves of deletion mutants from a 1042 WT background.**

**Supplementary Table S1**

| **Primer name** | **Sequence (5’-3’)** | **Description** |
| --- | --- | --- |
| pHoss1_F | GGATTTACTCCTGGAGCTGGT | Amplification and sequencing of the MCS of the temperature-sensitive pHoss1 vector |
| pHoss1_R | CCATTATGATTCTTCTCGCTTCCG |  |
| gttB_pHoss1_F | TTTCGAATTCTAATCATGCGGACTATCGACT | Amplification of the *gttB* flanking regions from WSLC 1042, containing restriction sites for insertion into the pHoss1 MCS |
| gttB _pHoss1_R | TTTGTCGACTATCGAATAAAATAGCTCGGGATTT |  |
| gtlB_pHoss1_F | TTTCGAATTCTGTTTTGCGTGAAGGGGA | Amplification of the *gtlB* flanking regions from WSLC 1042, containing restriction sites for insertion into the pHoss1 MCS |
| gtlB_pHoss1_R | TTTGTCGACTTGTCTCTCTATTGTCAGATGTTAC |  |
| gtlB_check_F | CATCTCTACATCGCCACC | Primers annealing outside the *gtlB* flanking regions to evaluate proper genetic deletion |
| gtlB_check_R | TTCCCGTTTGCGTCAGTT |  |
| gttB _check_F | GTGTCGTGTCTATTGATG | Primers annealing outside the *gttB* flanking regions to evaluate proper genetic deletion |
| gttB _check_R | GATAGAGTTTCGCACAAG |  |
| pIMK_fwd | TCCAATTCGCCCTATAGTG | Amplification of the pIMK2 vector for purposes of Gibson assembly, used for overexpression of the *gttB* gene in the 1042Δ*gttB* background |
| pIMK_rev | GCTCCACCGCGGTGGCGG |  |
| gttB c_fwd | ggccgccaccgcggtggagcCTCGTTTTACTATTGAAGAGAATG | Amplification of the *gttB* gene for purposes of insertion via Gibson assembly into the pIMK2 vector |
| gttB c_rev | tcactatagggcgaattggaTCATCTATTATCTCCAGTTTTC |  |
| Gp19_F | TTTGAGCTCGATTTAAAAAAATGGCAAGACCCA | Amplification of the gp19 protein from phage A500 for insertion into the SacI/SalI restriction sites into pHGFP |
| Gp19_R | TTTGTCGACTCATGTTCTAACCACTCCC |  |

**Supplementary Table S2**

| **Strain** | **Description** | **Source** |
| --- | --- | --- |
| WSLC 1042 | *L. monocytogenes* serovar 4b | ATCC 23074;  GenBank CP007210.1 |
| 1042Δ*gttA* | Deletion of *gttA* (AX24_RS02630) | Sumrall et al., 2019 |
| 1042Δ*gttB* | Deletion of *gttB* (AX24_RS02635) | This study |
| 1042Δ*gtlB* | Deletion of *gtlB* (AX24_RS00400) | This study |
| 1042Δ*gttB*::pIMK2(*gttB*) | Deletion mutant overexpressing *gttB* from P_help_ promoter | This study |
|  |  |  |
| Bacteriophage A500 | Serovar 4b-specific host range, temperate | Zink et al., 1992 |
| Bacteriophage PSA | Serovar 4b-specific host range, temperate | Zimmer et al., 2003 |

**Supplementary Table S3**

| **Plasmid** | **Description** | **Source** |
| --- | --- | --- |
| pHGFP | pQE30 with hGFP cloned into the BamHI/SacI sites | Schmelcher et al., 2010 |
| pA500-RBP-GFP | pHGFP expressing the gp19-GFP fusion protein with gal-WTA specificity | This study |
| pHoss1 | Encodes amp and ery resistance | Abdelhamed et al., 2015 |
| pGttA KO | *gttA* flanking regions (500bp) in pHoss1 | Sumrall et al., 2019 |
| pGttB KO | *gttB* flanking regions (500bp) in pHoss1 | This study |
| pGtlB KO | *gtlB* flanks in pHoss1 | This study |
| pIMK2 | Integrative vector for purposes of overexpression via the P_help_ promoter | Monk et al., 2008 |
| pIMK2(*gttB*) | pIMK2 vector for overexpressing *gttB* | This study |
